# Care of the Feet

**Published:** 1856-06

**Authors:** 


### CARE OF THE FEET.

One evening in Boston, just as *Washington Alston*, the Painter, was approaching the door of a dwelling, where a splendid party had assembled, he suddenly stopped short and said to his friend,

"I cannot go in."

Nonsense! why not?

"I have a hole in one of my stockings."

Pshaw, man, nobody knows it.

"But I do," said the celebrated Artist, as he turned on his heel and left his friend in doubt, whether to swear or laugh out-right.

A long time ago, "when you and I were boys," reader, when dead people were brought in and thrown down upon the floor of the dissecting room, just as indifferently as a brawny butcher throws down a great big pig to dissect into sausage meat, ham and spare-rib, and just as nude, except the face, which alone tells in the recent subject, that the man is dead, we used as a past-time, while the lecturer was calling over long Latin and Greek names, as dry as a fence-rail, and as hard, to be cogitating in our own minds, what was the position of that body when in life, what its relative standing in society. Somehow or other we fell on the feet, as the most reliable indicator, especially, if the appearance of the body as to plumpness, indicated sudden death. Now and then, the well trimmed toe-nail, its freedom from collections under it, and in every other spot from toe-nail to ankle, scrupulously clean ; these showed full well, that the poor body so ruthlessly treated now, was tenanted but a few hours before, by a spirit of purity, refinement and elevation, or had friends around it in the last sad hours of life, who merited such a character; and it was impossible to withhold our sympathy and respect for that lump of lifeless clay. At other times, the feet would be found in so filthy a condition, as to excite within us sentiments of the most irrestrainable disgust and contempt, and we felt as if the spirit which had so recently left that tenement was as foul and low as bestiality could make it.

On a beautiful November afternoon, away back yonder in the Forties, we had just stepped ashore on the Levee at New Orleans after a ten days' journey from Louisville, and hurrying along down the water's edge, a few yards from the shore, in the direction of the Post-Office, thinking of how many letters we would find there from absent friends, and kindred, and patients, we were aroused from our reverie by a tremendous concussion and noise ; the first glance was upward at the sky, filled with innumerable objects of every size and description ; they had scarcely got high enough to take their turn downwards, and the first thought, that miracle of instinct was, could we by any rate of locomotion put ourselves beyond the point at which the falling articles would strike the earth ; we looked again, and thought we could; if any individual ever "*heeled it*" in double quick time, it was the writer of this article; every hair of the head and body seemed to stand on end, a chill thrilled through the whole frame at every successive step, we felt an expectation of an instantaneous crush to the earth! Oh, how long that race for life seemed, for we were not forty yards from the *Louisiana*, at the moment of explosion. Not a single thing touched us, although we heard many pattering around us, apparently as thick as hailstones. In an instant we stood still, why, we cannot say, it was instinctive, not rational, and as soon as the sound of falling ceased, we turned to the scene of disaster; just as we turned, a poor young fellow passed us, scarcely able to limp along, and the next instant, was a full grown man, flat on his back, without one atom of injury except he had no head; the back-bone just protruded a little above the line of the shoulder. In that instant of time, some eighty-one persons if we remember well, were hurried into eternity. Some lingered a moment and died, others laid a long time and no aid came to them. The whole surface of the levee was covered with bits of human bones, and joints, and flesh, and hair, and parts of clothing: a piece of boiler weighing perhaps a thousand pounds, struck a bale of cotton, cutting a mule in two, and shivered a cast iron awning post, some four hundred feet from the ill-fated steamer. As litter after litter passed by us towards the hospital and town, bearing its blackened, mutilated, groaning, dying occupants, a resolution suddenly formed itself in our mind, as apparently foreign to scenes like these, as it was possible to be—that as long as we lived, we never would if alone, put our foot on a steamer or rail-car, except in our best clothing, and the whole body in as unexceptionable condition as razor, and soap, and water could make it. Now, why? The argument ran itself out in our mind as follows:—"If in that terrible hour, I had been bereft of all sense, the attention shown me, and the place assigned me in a private house or public hotel, or large hospital, would have depended, to a considerable extent, on the character of personal belongings." This is a thought which will bear maturing by all travellers.

Therefore, reader, if you would secure more marked attention from your physicians or nurses in times of sudden calamities and terrible mutilations of body, a clean person, a clean foot, would not be a despised passport.

The feet should be soaked in warm water, for at least twenty minutes, twice a week, and at the same time, rubbed and scrubbed with a brush and soap. Besides this, if they were dipped in cold water of mornings, ankle deep, both in at once, for a single minute, winter and summer, having them vigorously and briskly rubbed all the time they are in, then wiped dry and a walk taken, or held to a fire until perfectly warmed, the skin of the feet would be kept in a soft, cleanly, pliable condition, the circulation about them would be vigorous, and the result would be, in many instances, that corns and callosities would almost cease to trouble you; coldness of feet would, to a considerable extent, be removed, and "*taking cold*" would not occur once, where it now occurs a dozen times; for it is through the feet, that many of our most serious ailments come. In addition, let us suggest, that one of the most useful of habits, as well as agreeable, during all the season of the year, in which fires are kept burning, let the last operation preceding getting into bed be, holding the naked foot to the fire, for ten or fifteen minutes, rubbing with the hands all the time, until most thoroughly dry and warm. A good anodyne that.

